# Aryl Hydrocarbon Receptor Activation Ameliorates Acute Respiratory Distress Syndrome through Regulation of Th17 and Th22 Cells in the Lungs

**DOI:** 10.1128/mbio.03137-22

**Published:** 2023-02-21

**Authors:** Bryan Latrell Holloman, Alkeiver Cannon, Kiesha Wilson, Prakash Nagarkatti, Mitzi Nagarkatti

**Affiliations:** a Department of Pathology, Microbiology, and Immunology, University of South Carolina School of Medicine, Columbia, South Carolina, USA; University of Oklahoma Health Sciences Center

**Keywords:** ARDS, aryl hydrocarbon receptor, Th22 cells, lung inflammation

## Abstract

Acute respiratory distress syndrome (ARDS) is triggered by a variety of insults, including bacterial and viral infections, and this leads to high mortality. While the role of the aryl hydrocarbon receptor (AhR) in mucosal immunity is being increasingly recognized, its function during ARDS is unclear. In the current study, we investigated the role of AhR in LPS-induced ARDS. AhR ligand, indole-3-carbinol (I3C), attenuated ARDS which was associated with a decrease in CD4^+^ RORγt ^+^IL-17a^+^IL-22^+^ pathogenic Th17 cells, but not CD4^+^RORγt ^+^IL-17a^+^IL-22^−^ homeostatic Th 17 cells, in the lungs. AhR activation also led to a significant increase in CD4^+^IL-17a^−^IL-22^+^ Th22 cells. I3C-mediated Th22 cell expansion was dependent on the AhR expression on RORγt^+^ cells. AhR activation downregulated miR-29b-2-5p in immune cells from the lungs, which in turn downregulated RORc expression and upregulated IL-22. Collectively, the current study suggests that AhR activation can attenuate ARDS and may serve as a therapeutic modality by which to treat this complex disorder.

## INTRODUCTION

ARDS is a form of respiratory failure that is caused by a variety of insults, including pneumonia, sepsis, trauma, and certain viral infections, including SARS-CoV2, which caused the COVID-19 pandemic (1). It is estimated that approximately 33% of hospitalized COVID-19 patients develop ARDS and that nearly 75% of such patients admitted to the intensive care unit have ARDS (2). It is difficult to treat ARDS, and because of this difficulty, nearly 37% of ARDS patients die annually (3). ARDS is characterized by pulmonary inflammation that leads to capillary endothelial injury, diffuse alveolar damage, and poor oxygenation (4). AhR is a ligand-activated transcription factor that acts as an environmental sensor to regulate inflammation. While its role in ARDS has not been well-studied, AhR deficiency can lead to hyperoxia-induced lung inflammation and damage, whereas the activation of AhR mitigates the effect of hyperoxia (5, 6). However, the mechanisms behind the therapeutic efficacy of AhR remain poorly defined.

The AhR is expressed in various immune cells in the lungs, including Th17 and Th22. The production of IL-17a by Th17 cells provides protection against extracellular pathogens and the clearance of bacteria, and it also plays a part in homeostatic physiological processes in the lungs; however, Th17 cells have also been demonstrated to exacerbate lung injuries (7, 8). Interestingly, two subsets of Th17 have been identified, and this may explain their contradictive behavior. Homeostatic Th17 RORγt ^+^IL-17^+^IL-22^−^ cells do not induce tissue inflammation and are considered immunosuppressive. However, under inflammatory conditions, homeostatic Th17 RORγt^+^IL-17a^+^IL-22^−^ cells are converted into Th17 RORγt^+^IL-17a^+^IL-22^+^ cells, which are recognized as pathogenic Th17 cells that cause tissue damage (9–11). While the pathogenic role of Th17 cells has been identified in ARDS, studies looking into the mechanisms that are involved in the conversion of homeostatic Th17 RORγt ^+^IL-17a^+^IL-22^−^ cells to Th17 RORγt ^+^IL-17a^+^IL-22^+^ cells during lung injuries are limited (12). Furthermore, Th22 cells also play a role in lung homeostasis by producing IL-22, a cytokine that helps maintain the lung barrier, the integrity of intestinal and lung epithelial cells, and the production of antimicrobial peptides (7, 13–16). Conversely, despite its protective role, IL-22 is involved in the development of chronic inflammatory diseases (17–20), implicating this cytokine as being multifaceted and as being involved in both proinflammatory and anti-inflammatory conditions. Interestingly, in several inflammatory disorders, IL-17a and IL-22 have contradicting responses. In uveitis and colitis, IL-17a levels are positively allied with disease severity. At the same time, IL-22 is negatively correlated with disease severity, and the deletion of IL-22 results in increases in inflammatory mediators, disease severity, and Th17 lymphocytes during inflammation (21, 22).

Furthermore, Th17 development is regulated by RORγt and by the aryl hydrocarbon receptor (AhR) (23). Th22 cell differentiation is reliant on the AhR and partially depends on RORγt expressing cells (24). The activation of the AhR alone leads to the production of IL-22 by intestinal leukocytes, and it can also act synergistically with the transcription factor RORγt, a cell signature of Th17 cells (25, 26). Quintana et al. (2008) reported that the transcription of IL-22 is induced through the cooperation of AhR with RORγt, which leads to the production of IL-22 (27). In the lungs, IL-22 in the presence of IL-17a promotes lung inflammation, whereas IL-22 protects against lung injury in the absence of IL-17a (28, 29). In the gut, the AhR-IL-22 axis is important in tissue maintenance (25). Interestingly, AhR-deficient mice have increased intestinal pathogenic Th17 cells, but the administration of IL-22-Ig reduced the cell frequency of the pathogenic Th17 cells (25). Ironically, Th22 cells produce high levels of IL-22 without the secretion of IL-17, which is controlled by the AhR (30, 31). Furthermore, Th17 cells and Th22 cells exhibit a bidirectional phenotypic flexibility that may be linked to the relationship between AhR, RORγt, IL-22, and IL-17a (30). Therefore, a feedback loop that is controlled by IL-22 and the AhR may regulate the levels of pathogenic Th17 cells through the AhR ligand-mediated production of IL-22 from Th22 cells. Targeting the Th17/Th22 axis may be beneficial in treating inflammatory diseases. In addition, the AhR also protects mice against hyperoxic lung injury by regulating the expression of RelB, a component of the nuclear factor-kappaB (NF-κB) protein (18). Interestingly a RelBAhRE DNA binding site during LPS stimulation is necessary to activate the IL-22 promoter in bone marrow macrophages (32). However, the mechanism behind AhR inducing IL-22 production in T helper cells during ARDS is not well-defined.

Indole-3-carbinol (I3C) is a natural indole compound that is found in cruciferous vegetables. I3C has various therapeutic benefits, including anti-inflammatory, antimicrobial, and immunomodulatory properties. The immunomodulatory capabilities of I3C are driven by its role as an AhR ligand (15, 33). The AhR is located on various immune and epithelial cell types. The administration of I3C was shown to decrease Th17 cells while increasing the expression of IL-22-secreting lymphocytes in 2,4,6-trinitrobenzene sulfonic acid-induced colitis models (15). In addition, I3C has been shown to regulate microRNAs (miRNAs) that are associated with inflammation, Th17 cells, and the production of antimicrobial peptides (14, 15).

In the current study, we investigated the role of AhR activation by I3C on the Th17/Th22 axis by examining the roles of miRNAs as well as their involvement in the regulation of RORγt ^+^IL-17a^+^ IL-22^−^ and RORγt ^+^IL-17a^+^IL-22^+^ Th17 cells and Th22 cells (IL-17a-IL-22^+^) during LPS-mediated ARDS. Our studies demonstrated that I3C attenuates ARDS through the downregulation of Th17 RORγt ^+^IL-17a^+^IL-22^+^ cells and the upregulation of Th22 IL-17a-IL-22^+^ lymphocytes. Furthermore, we found that the I3C-mediated shift in the Th17 RORγt ^+^IL-17a^+^IL-22^+^ cells and Th22 IL-17a-IL-22^+^ lymphocytes was dependent on the AhR on RORγt^+^ cells and was orchestrated by miRNA-29b-2-5p, the role of which in ARDS has not been studied previously. We found that the expression of miRNA-29b-2-5p was downregulated in mice with ARDS following treatment with I3C. Interestingly, a miRNA-29b-2-5p inhibitor increased IL-22 and AhR gene expression, but it decreased RORc expression in lung mononuclear cells, whereas a miRNA-29b-2-5p mimic had the opposite effects. Together, these findings open the possibility of targeting the Th17/Th22 axis and miRNA-29b-2-5p for the attenuation of ARDS.

## RESULTS

### An I3C treatment upholds lung structure organization and restores pulmonary functions during ARDS.

**FIG 1:**
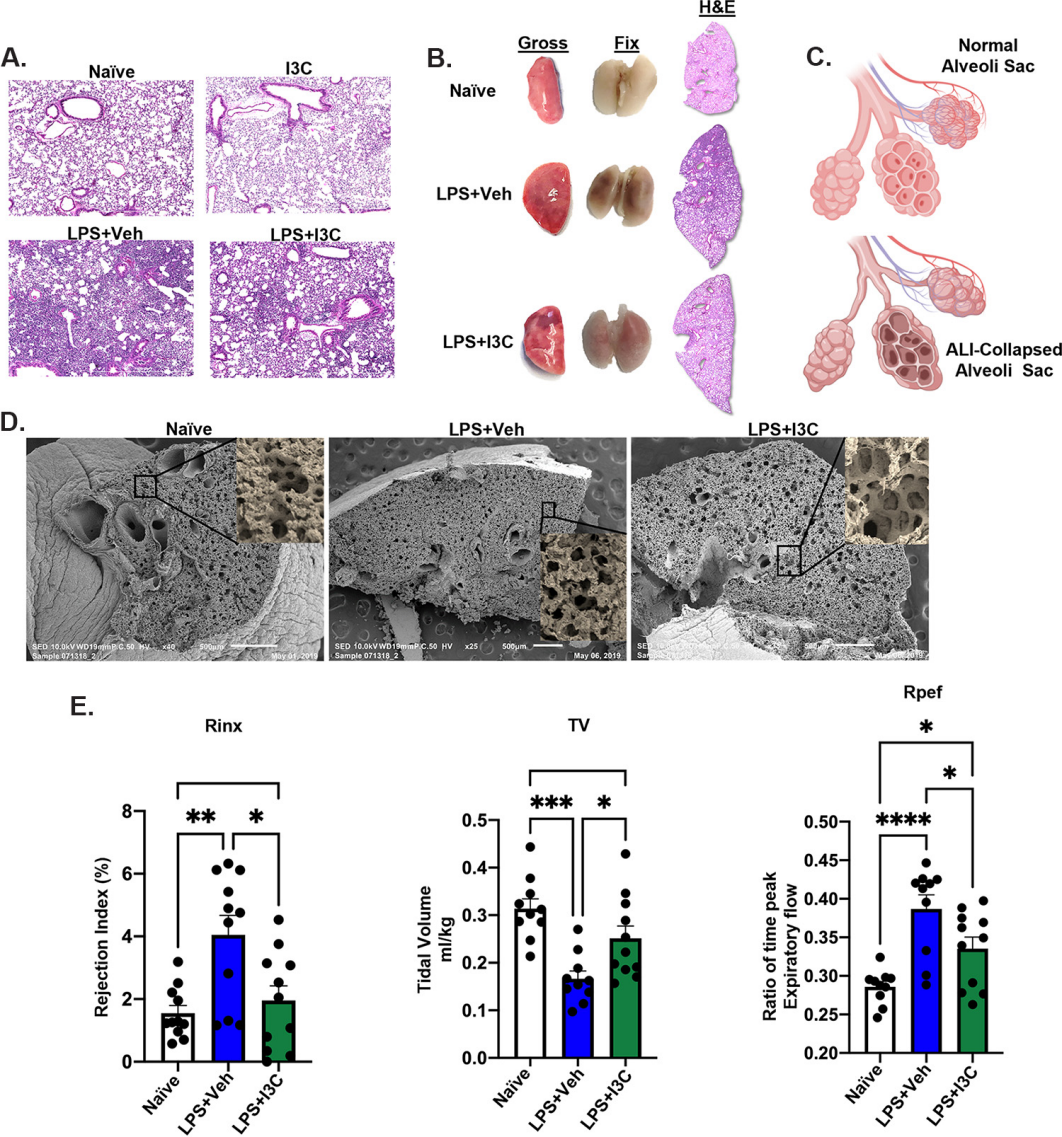
I3C treatment restores basal pulmonary functions, reduces lung tissue damage, and prevents lung remodeling following LPS-mediated ARDS induction in C57BL/6J mice. Mice were untreated (naive), treated with LPS (2 mg/kg) + Veh intranasally, or treated with LPS + I3C with the I3C being given 3 h after the LPS. After 24 h, each mouse underwent a plethysmography test. The mice were euthanized at 48 h, and their lungs were processed for analysis. (A) Hematoxylin and eosin-stained sections (4×). (B) Gross appearance of a left lung lobe, fixed whole lungs, and hematoxylin and eosin-stained montage sections of C57BL/6J mice. (C) Schematic of normal and diseased mice alveolar sacs. (D) Scanning electron microscopic images of the left lung lobes of mice. The box indicates an alveolar sac (collapsed alveoli in LPS + Veh). (E) Bar graph representation of the ratio of time expiratory flow (Rpef), rejection index (RINX), and tidal volume (TV) (*n* = 10 to 11). The data are depicted as the mean ± the standard error of the mean (SEM). Statistical significance was tested via a two-tailed, one-way analysis of variance (ANOVA). The following values relate to panel E. RINX: *, *P* = 0.0104; **, *P* = 0.0022; TV: *, *P* = 0.0250; ***, *P* = 0.0002; Rpef: *, *P* = 0484; *, *P* = 0.03897; ****, *P* < 0.0001.

We evaluated lung structures in mice with LPS-mediated ARDS using light and scanning electron microscopy. We observed an influx of infiltrating immune cells in the lungs of mice treated with LPS+Veh, compared to naive mice (Fig. 1A). In contrast, mice treated with LPS+I3C showed a significant decrease in cellular infiltration in their lungs, compared to those of the LPS+Veh group (Fig. 1A). Alveolar and bronchiolar epithelial hyperplasia accompanied the influx of immune cells in the LPS+Veh-treated animals. In contrast, I3C blocked the epithelial hyperplasia formation (Fig. 1A). To examine the tissue damage formation in the lung lobes resulting from ARDS as well as the wound healing response, we studied the gross anatomy of the lungs, fixed lungs, and hematoxylin and eosin (H&E) montage. Examining the gross anatomy of the left lung lobes in LPS+Veh-treated mice and in mice treated with LPS+I3C, we noted that treatment with I3C caused a decrease in the size of the lungs. However, there was no significant difference in wet-dry weight in the LPS+Veh-treated mice versus the LPS+I3C-treated group. Interestingly, after fixing the lungs, the lungs of the LPS+Veh-treated animals showed dark scar tissue that was associated with necrosis (anoxia), whereas the lung tissues of the LPS^+^ I3C-treated mice were pink in appearance, which was associated with tissue repair. The H&E montage images showed that I3C decreased the epithelial hyperplasia across the lung lobe (Fig. 1B). Furthermore, the schematic shows the difference in appearance in the alveolar sac during lung hemostasis and during lung injury (Fig. 1C). We observed collapsed alveolar sacs in diseased animals, but the integrity of the alveolar sacs was reversed following the I3C treatment (Fig. 1D). Together, these findings suggest that I3C reduces lung inflammation and helps maintain lung structure organization during LPS-induced ARDS.

Next, we investigated whether I3C helps restore lung functionality in LPS-treated, diseased animals. Using whole-body plethysmography, we found that I3C restored several lung functions back to basal levels. I3C treatment decreased the percentage of rejected breaths during the lung functional testing duration (RINX) and increased the amount of air moving in and out of the lungs during each respiratory cycle (TV) in diseased mice. In addition, I3C restored the ratio of peak expiratory flow (Rpef) functionality of diseased mice to basal levels (Fig. 1E). Taken together, these data suggest the beneficial role of I3C in alleviating acute lung injuries.

### The I3C treatment of diseased mice decreases pathogenic Th17 cells and increases the Th22 cell population in the lungs during ARDS.

**FIG 2:**
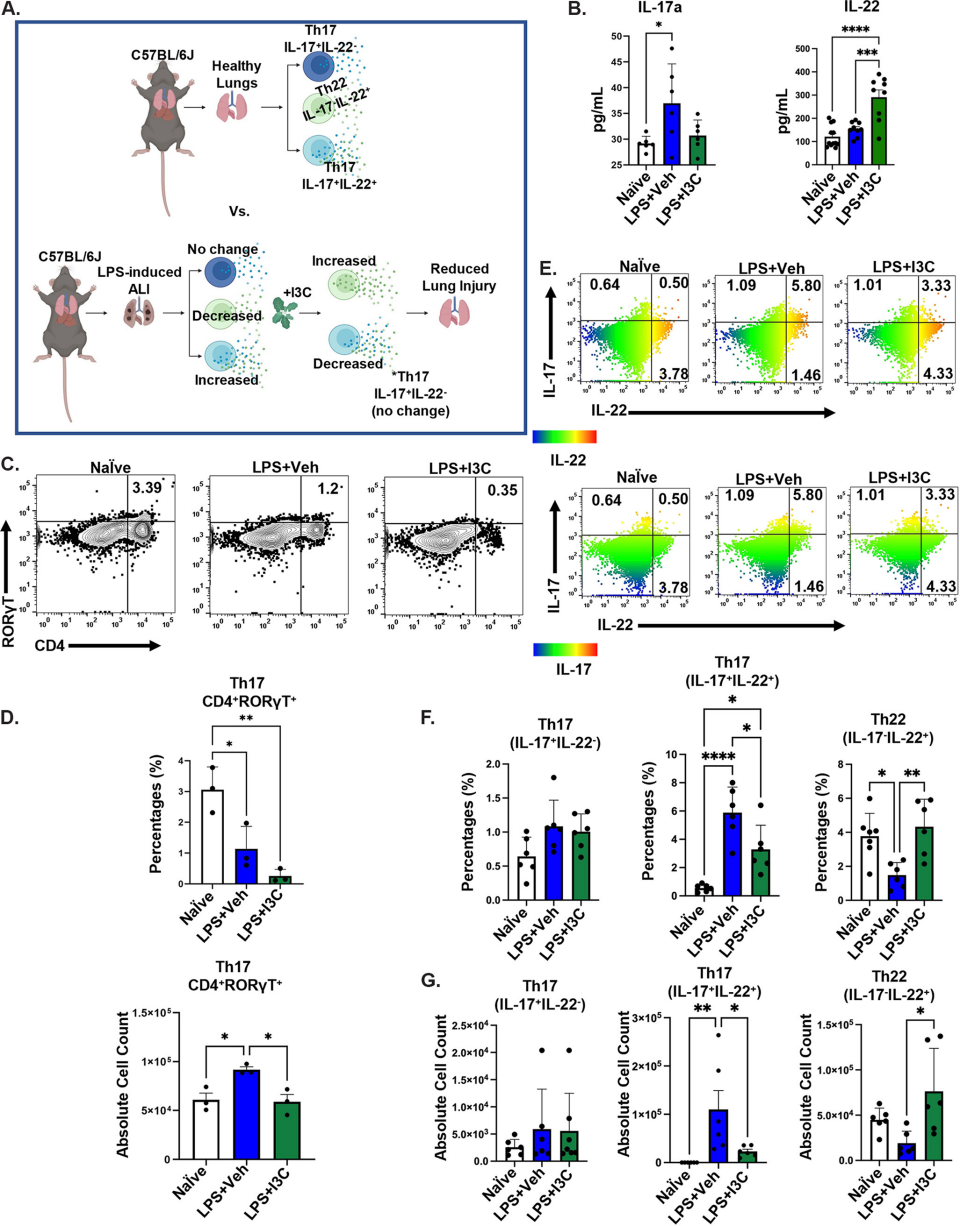
Th22 cells and IL-22 production are increased following the I3C treatment of LPS-induced ARDS. Mice were administered with LPS to induce ARDS, and they were treated with I3C as described in the legend of Fig. 1. The mice were euthanized at 48 h, and their lungs were processed for analysis. (A) Experimental design schematic of LPS-mediated ARDS in C57BL6/J mice treated with I3C. (B) IL-17a and IL-22 protein levels in bronchial lavage fluid were detected via enzyme-linked immunosorbent assay (*n* = 6 to 12). (C) Representative contour flow cytometry plots of CD4^+^ RORγT^+^ cells. (D) Percentages and absolute cell numbers (*n* = 3) of CD4^+^ RORγT^+^ T cells. (E) Representative flow cytometry heat map plots of cells stained for IL-17a and IL-22. (F and G) Percentages and absolute cell numbers, respectively, of IL-17a- and IL-22-secreting T cells (*n* = 6). The data are depicted as the mean ± the standard error of the mean (SEM). Statistical significance was tested via a two-tailed, one-way ANOVA. In panel B: *, *P* = 0.0330; ****, *P* < 0.0001; ***, *P* = 0.0002. In panel D: *, *P* = 0.0196; **, *P* = 0.0032; *, *P* = 0.0284; *, *P* = 0.0217. In panel F: ****, *P* < 0.0001; *, *P* = 0.0136; *, *P* = 0.0184; *, *P* = 0.0149; *, *P* = 0.0041. In panel G: **, *P* = 0.0097; *, *P* = 0.0398.

Next, we studied the involvement of Th17 and Th22 during homeostasis and during a pathogenic state, as these subsets of T cells are involved in lung epithelium maintenance, reepithelization, and lung remodeling during ARDS. Toward this, Mononuclear cells (MNCs) were isolated from whole lung tissue, and we used ELISA and flow cytometry assays to identify Th17/Th22 subsets (Fig. 2A). The IL-17a protein levels were increased in the bronchial alveolar lavage fluid (BALF) of LPS+Veh-treated diseased animals, whereas the IL-22 protein levels were not significantly altered in these animals, compared to naive mice. Interestingly, decreased levels of IL-17a and elevated levels of IL-22 were found in the BALF of the LPS+I3C-treated animals, compared to those found in the LPS+Veh-treated animals (Fig. 2B). In addition, the CD4^+^RORγt^+^ total cell numbers were significantly increased in the LPS+Veh group versus the naive group, and the treatment with I3C caused a decrease in these cells (Fig. 2C and D). We observed two distinct populations of Th17 cells, consisting of IL-17a^+^ IL-22^−^ and IL-17a^+^IL-22^+^ (Fig. 2E), which we termed homeostatic Th17 cells and pathogenic Th17 cells, respectively. Furthermore, there were no changes in the percentages or absolute numbers of the homeostatic Th17 cell population across treatment groups (Fig. 2F and G). On the other hand, the percentages and absolute numbers of pathogenic Th17 (IL-17a^+^IL-22^+^) were significantly increased in the LPS+Veh group, compared to the naive lungs (Fig. 2F and E), and the I3C treatment caused a significant decrease in these cells. ARDS also disrupted the population of Th22 cells (IL-17-IL-22^+^) in the lungs, resulting in a decrease in the percentages and absolute numbers of Th22 cells in the lungs of the LPS+Veh-treated, diseased animals (Fig. 2D and E). However, I3C led to a significant increase in Th22 cells in the lungs of ARDS animals (Fig. 2E–G). Together, these studies suggest that I3C decreases pathogenic Th17 cells during LPS-mediated ARDS without significantly affecting homeostatic Th17 cells. Also, I3C increased the levels of Th22 cells in the lungs during ARDS.

### The I3C treatment increased Th22 gene signatures in Th17 cells *in vitro*.

**FIG 3:**
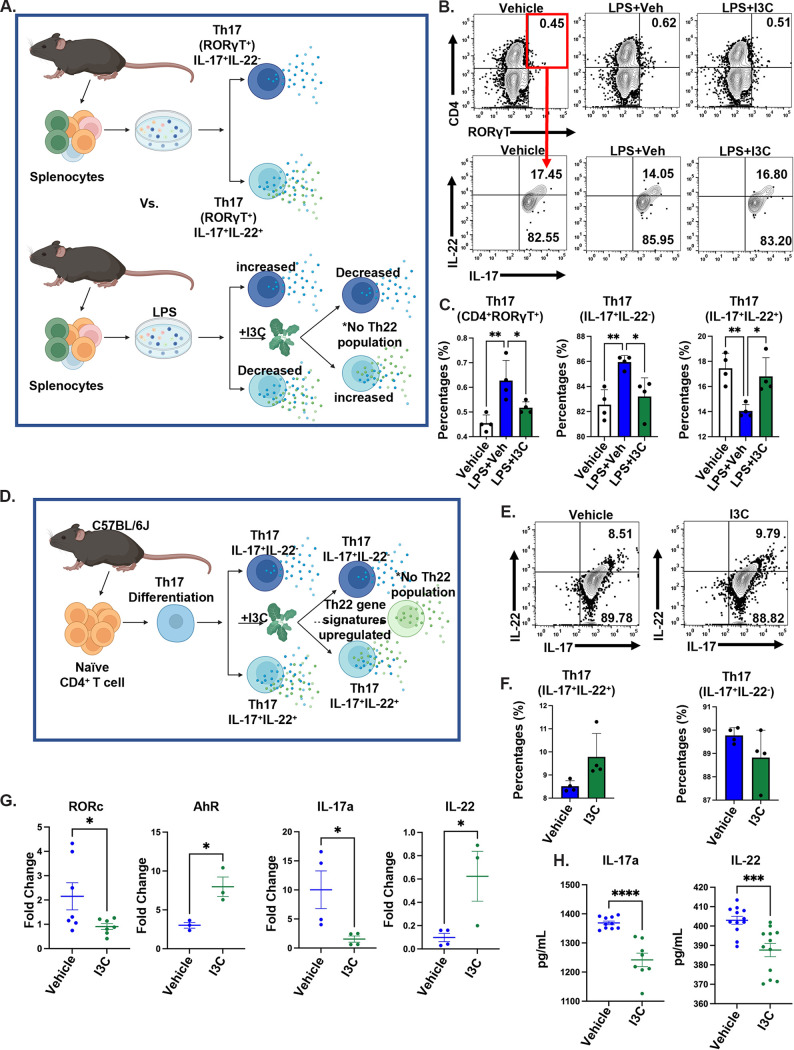
*In vitro* I3C-treatment increased Th22 gene signatures in Th17 cells. (A) Experimental design schematic of *in vitro* cultured splenocytes taken from C57BL6/J mice treated with vehicle, LPS+Veh, or LPS+I3C. Following treatment, the cells were cultured for 72 h before downstream analysis. (B) Representative contour flow cytometry plots of CD4^+^ RORγ^+^ cells, which were further analyzed for IL-22 and IL-17. (C) Bar graph of the percentages of CD4^+^ RORγ^+^, CD4^+^RORγ^+^ IL-17^+^ IL-22^−^, and CD4^+^ RORγ^+^ IL-17^+^ IL-22^+^ (Th17) cells from multiple experiments (*n* = 4). (D) Schematic of CD4^+^ T cells cultured under Th17 conditions and treated with vehicle or I3C. Following treatment, the cells were cultured for 72 h before downstream analysis. (E) Representative contour flow cytometry plots of CD4^+^ cells stained for IL-22 and IL-17 after culturing, as described in panel D. (F) Bar graph of the percentages of CD4^+^ IL-17^+^ IL-22^−^ and IL-17^+^ IL-22^+^ cells from multiple experiments (*n* = 4). (G) RORc, IL-17A, AhR, and IL-22 mRNA expression in polarized Th17 cells, as detected via RT-PCR (*n* = 3 to 7). (H) IL-17 and IL-22 protein levels in polarized heterogeneous Th17 cell culture supernatant, as detected via enzyme-linked immunosorbent assay (*n* = 8 to 12). The data are depicted as the mean ± the standard error of the mean (SEM). Statistical significance was tested via a two-tailed, one-way ANOVA or via a two-tailed, paired Student’s *t* test. In panel C: **, *P* = 0.0032; *, *P* = 0.0392; **, *P* = 0.0058; *, *P* = 0.0193; **, *P* = 0.0058; *, *P* = 0.0193. In panel G: *, *P* = 0.0499; *, *P* = 0.0198; *, *P* = 0.0416; *, *P* = 0.0356. In panel H. ****, *P* < 0.0001; ***, *P* = 0.0008.

To investigate whether I3C was acting directly, we cultured splenocytes with and without LPS. Cultured cells were treated with either vehicle or 20 mM I3C (Fig. 3A). Contradictory to our *in vivo* studies, the *in vitro* LPS+I3C group showed a significant decrease in CD4^+^RORγt ^+^IL-17a^+^IL-22^−^ Th17 cells and increased CD4^+^RORγt^+^IL-17a^+^IL-22^+^ Th17 cells, compared to the LPS+Veh group (Fig. 3B and C). Interestingly, we did not observe a CD4^+^IL-17-IL-22^+^ (Th22) cell population in the cultures. To further investigate the effect of I3C on the Th17 cell subsets, we performed a Th17 polarization assay using naive CD4^+^ T cells that were isolated from spleens in the presence of either vehicle or I3C (Fig. 3D). It was revealed that, phenotypically, I3C did not affect the Th17 (CD4^+^IL-17a^+^IL-22^−^ and CD4^+^IL-17a^+^IL-22^+^) cells (Fig. 3E and F). However, I3C altered the gene signatures of the Th17 cells. I3C downregulated the expression of RORc and IL-17a in the Th17 cells while upregulating the expression of AhR and IL-22, which are gene signatures of Th22 (Fig. 3G). I3C decreased the IL-17a protein levels in the Th17 cultures but decreased IL-22 (Fig. 3H). The decrease in IL-22 in the culture supernatants (Fig. 3H), while an increase was seen when using RT-PCR (Fig. 3G), can be explained by the fact that in the former, cells may use IL-22 that is secreted.

### The I3C-mediated increase in the Th22 cell population and decrease in the pathogenic Th17 cell population are dependent on IL-22.

**FIG 4:**
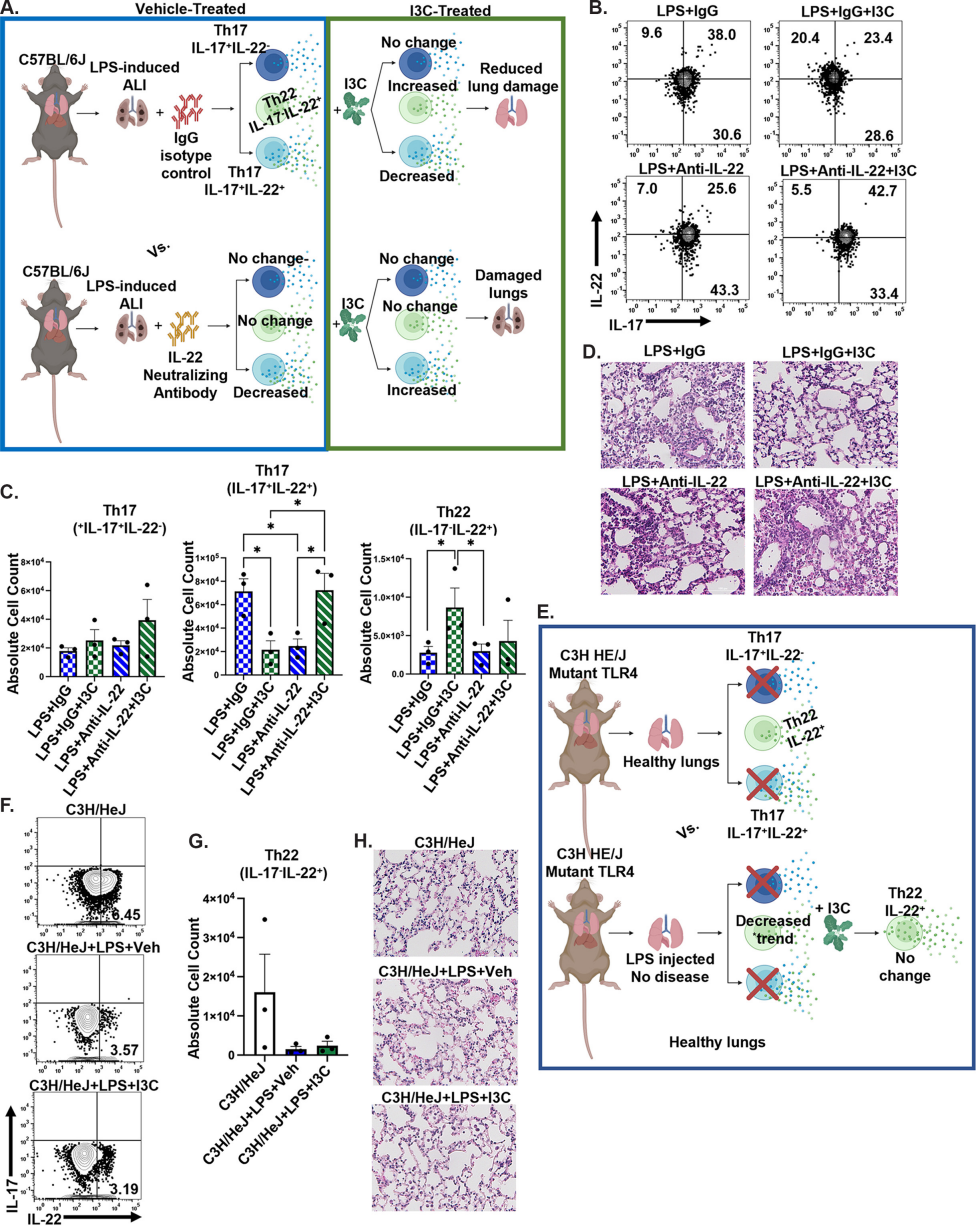
The I3C-mediated increase in the Th22 cell population is dependent on an increase in IL-22. (A) Experimental design schematic of LPS-administered C57BL6/J mice treated with an anti-IL-22 neutralizing antibody or an IgG isotype as a control. The mice were euthanized at 48 h, and their lungs were processed for analysis. (B) Representative flow cytometry contour plots of cells that were double-stained for IL-17 and IL-22 populations in LPS+Veh and LPS+I3C mice that were treated with an IgG control or an IL-22 neutralizing antibody. (C) Absolute cell counts of CD4^+^ Th17 and Th22 cells (*n* = 3). (D) Hematoxylin and eosin-stained sections (20×). (E) Experimental design schematic of naive C3H/HeJ versus LPS+Veh versus LPS+I3C groups of mice. (F) Representative contour flow cytometry plots of CD4^+^ IL-22^+^ and CD4^+^ IL-17^+^ cells. (G) Absolute cell count of IL-22 secreting Th22 cells (*n* = 3). (H) Hematoxylin and eosin-stained sections (20×). Data were depicted as the mean ± the standard error of the mean (SEM). Statistical significance was tested via a two-tailed, one-way ANOVA. In panel C: *, *P* = 0.0351; *, *P* = 0.0478; *, *P* = 0.0321; *, *P* = 0.0436; *, *P* = 0.0491; *, *P* = 0.0487.

To determine whether the IL-22 protein level and IL-22 signaling play a role in the generation of Th22 cells during ARDS, we performed an IL-22 neutralization experiment using antibodies against IL-22 (Fig. 4A). Interestingly, neutralizing IL-22 *in vivo* (LPS+anti-IL-22) had no significant effect on the Th17 cell subset (IL-17a^+^IL-22^−^) compared to the LPS+ IgG group (Fig. 4A and C). However, the anti-IL-22 treatment downregulated pathogenic Th17 IL-17a^+^IL-22^+^ cells (LPS+anti-IL-22 versus LPS+ IgG). Moreover, the I3C-mediated downregulation of the Th17 IL-17a^+^IL-22^+^ cells was reversed following an anti-IL-22 antibody treatment. These data suggest that the effect of I3C to decrease pathogenic Th17 IL-17a^+^IL-22^+^ cells in LPS-treated mice is dependent on the presence of IL-22. Similarly, the ability of I3C to induce Th22 cells (IL-17a-IL-22^+^) following LPS administration was also dependent on IL-22, as a treatment with anti-IL-22 antibodies (Abs) reversed this effect of I3C (Fig. 4B and C). Importantly, lung histopathology showed that neutralizing IL-22 prevented the I3C-mediated attenuation of lung inflammation and lung structure damage (LPS+IgG+I3C versus LPS+anti-IL-22+I3C) (Fig. 4D). Together, these data demonstrate that IL-22 plays a beneficial role by suppressing lung injury in LPS-mediated ARDS and that I3C acts via the induction of IL-22.

We also observed that mice mutant in toll-like receptor 4 (C3H/HeJ) did not develop ARDS following LPS exposure and did not develop Th17 (IL-17a^+^IL-22^−^ and IL-17a^+^IL-22^+^) cells (Fig. 4E–H). Although we were able to detect Th22 cells, these cells were not significantly altered across the treatment groups (Fig. 4G). Lung histopathology showed no changes in lung structure across treatment groups (Fig. 4H) This suggested that I3C could induce Th22 cells only in wild-type mice with LPS-mediated ARDS *in vivo* but not in TLR4 mutant mice that were exposed to LPS.

### The AhR on RORgT^+^ cells is critical for the generation of Th22 following an I3C treatment during LPS-mediated ARDS.

**FIG 5:**
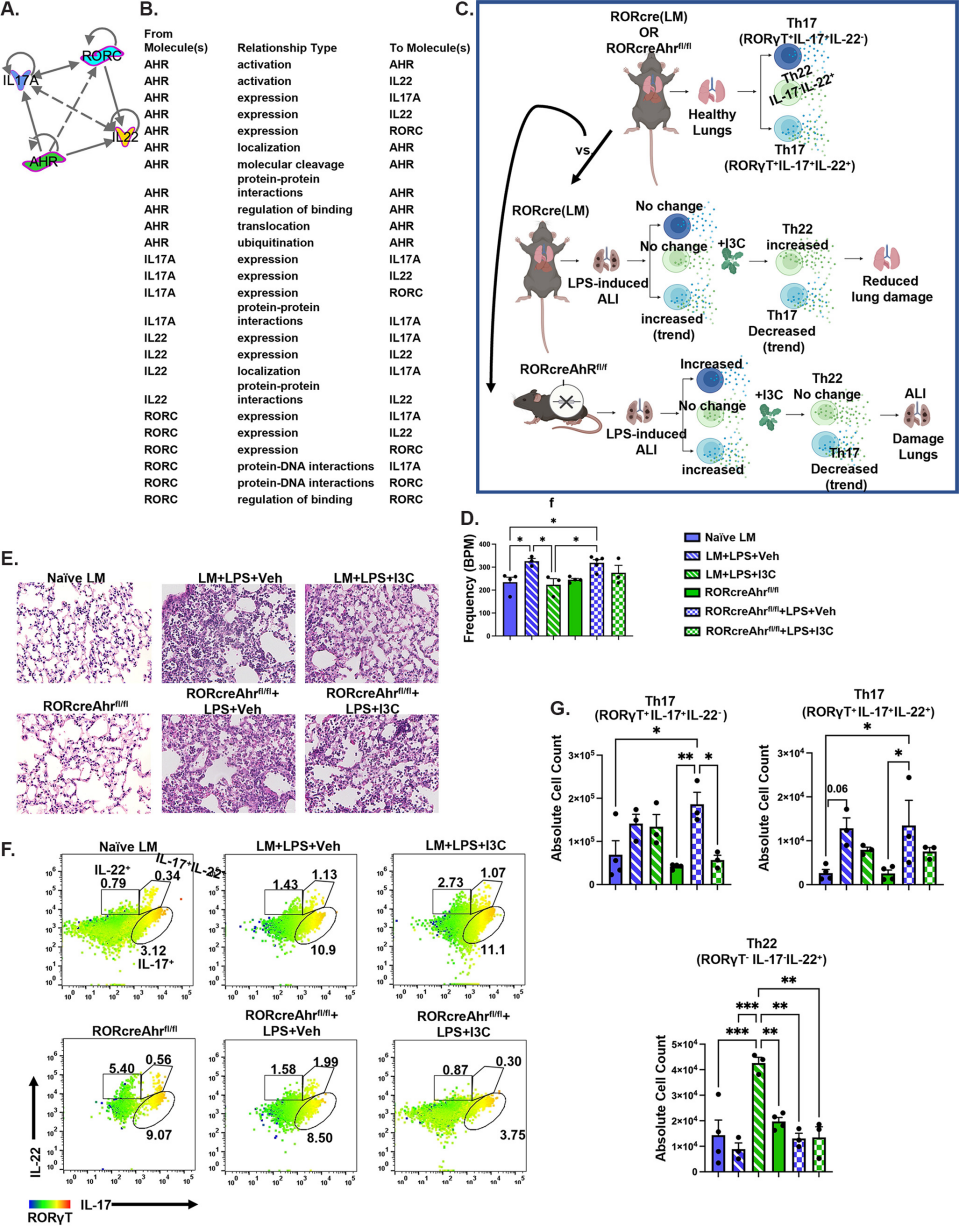
The AhR expression on RORgT^+^ cells is responsible for increasing the Th22 T cell subset following I3C treatment in LPS-exposed mice. (A and B) An Ingenuity Pathways Analysis shows the relationship between AhR, RORc, IL-17A, and IL-22. (C) Schematic of the experimental design. The mice were euthanized at 48 h, and their lungs were processed for analysis. (D) Bar graph representation of the frequency of breathing (f) (*n* = 4). (E) Hematoxylin and eosin-stained sections (20x). (F) Representative flow cytometry heat map plots of Th17/22 expressing cells (RORγT^+^IL-17^+^IL-22^−^, RORγT^+^IL-17^+^IL-22^+^, and RORγT-IL-17-IL-22^+^). (G) Absolute cell count of IL-17 and IL-22 expressing T cell subsets (*n* = 3 to 4). The data are depicted as the mean ± the standard error of the mean (SEM). Statistical significance was tested via a two-tailed, one-way ANOVA. In panel D: *, *P* = 0.0429; *, *P* = 0.0311; *, *P* = 0.0309; *, *P* = 0.0234; G. *, *P* = 0.0292; **, *P* = 0.0062; *, *P* = 0.0237; *, *P* = 0.0248; *P* = 0.0184; ***, *P* = 0.0010; ***, *P* = 0.0003; **, *P* = 0.0062; **, *P* = 0.0012; **, *P* = 0.0014.

Strikingly, producing-22-producing Th17 (IL-17a^+^IL-22^+^) and Th22 (IL-17a-IL-22^+^) cells exhibited a bidirectional phenotypic flexibility [34], which we speculate may stem from the interrelationship of the AhR, the RORγt transcription factor, IL-22, and IL-17. Interestingly, an Ingenuity Pathways Analysis (IPA) generated relationship connections between AhR, RORc, IL-17a, and IL-22 (Fig. 5A and B). Therefore, to confirm the roles of RORγt^+^ cells and AhR activation in the generation of Th22 cells and in the downregulation of pathogenic Th17 cells during ARDS, we used RORcreAhr^fl/fL^ mice and induced ARDS using LPS (Fig. 5C). RORcreAhr^fl/fL^ mice lack the AhR on their RORγt^+^ cells due to the knockout of the AhR gene in the RORγt^+^ cells. Littermate controls (LM) expressing AhR on RORγt^+^ cells were used to avoid any misinterpretations of our findings. Looking at the clinical parameters and breaths per minute taken by these mice, there were increased breaths per minute in the LPS-exposed animals treated with vehicle versus those of the naive animals in LM and RORcreAhr^fl/fL^ mice. The I3C treatment of the LPS-exposed animals restored the frequency of breaths per minute to the basal level in the LM mice; however, knocking out the AhR on RORγt^+^ prevented I3C-mediated recovery during ARDS (Fig. 5D). We also observed that AhR knockout in RORγt^+^ prevented the protective role of I3C in maintaining the integrity of the lung structure during ARDS (Fig. 5E). Thus, I3C-mediated lung repair and functionality recovery during ARDS are ablated when AhR is deficient in RORγt^+^ cells.

Furthermore, a flow cytometry analysis of RORcreAhr^fl/fL^ mice showed that the I3C-mediated induction of Th22 cell expansion was dependent on the AhR expression on RORγt^+^ cells during ARDS but that there were no changes in the pathogenic Th17 population in the RORcreAhr^fl/fL^+LPS group versus the RORcreAhr^fl/fL^+LPS+I3C group. Interestingly, ARDS in RORcreAhr^fl/fL^ mice caused an increase in Th17 RORγt ^+^IL-17a^+^IL-22^−^ and Th17 RORγt ^+^IL-17a^+^IL-22^+^ cells versus those of naive RORcreAhr^fl/fL^ mice and naive LM mice. However, I3C decreased the expansion of Th17 RORγt ^+^IL-17a^+^IL-22^−^ cells in ARDS-induced RORcreAhr^fl/fL^ mice, but there was not a significant difference in the Th17 RORγt ^+^IL-17a^+^IL-22^+^ cells when comparing the RORcreAhr^fl/fL^+LPS group versus the RORcreAhr^fl/fL^+LPS+I3C group (Fig. 5F and G). These data suggest that I3C-induced AhR activation on RORγt^+^ expressing cells controls tissue reparative Th22 induction during ARDS.

### The scRNAseq immune profiling of lung cells reveals that I3C decreases RORc expression while increasing the expression of IL-22 in C57BL/6 mice with ARDS.

**FIG 6:**
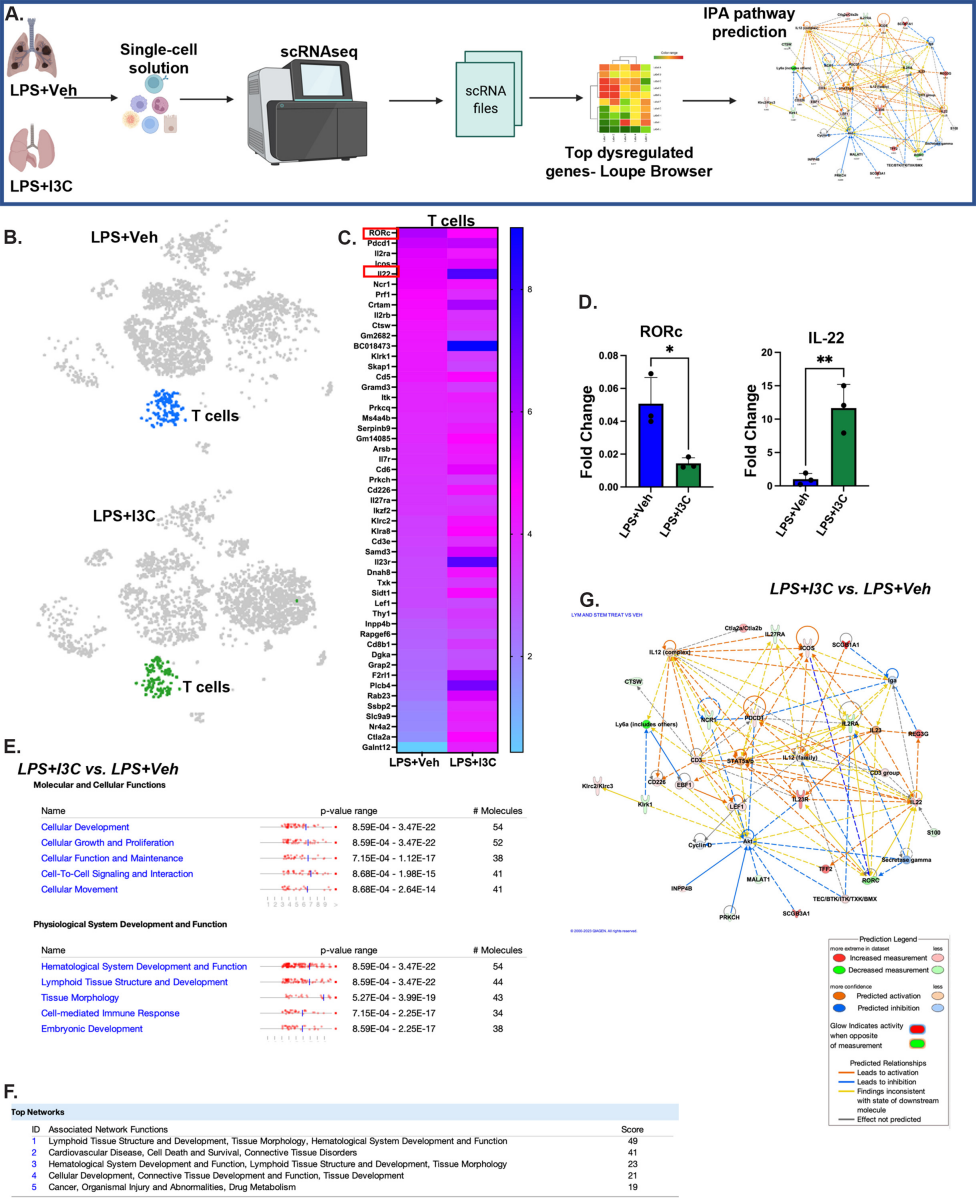
The scRNAseq immune profiling of lung-derived cells shows that I3C alters gene expression in T cells. Mice were treated with LPS+Veh or LPS+I3C, as described in the legend of Fig. 1. The mice were euthanized 48 h later, and their lungs were processed for an scRNAseq analysis. (A) Experimental design schematic to identify the pathways associated with dysregulated genes in lung cells following the I3C treatment of LPS-exposed mice, using 10× single-cell RNA sequencing and an IPA analysis. (B) Sample ID scRNAseq t-SNE of the T lymphocyte cluster. (C) Heatmap expression of top differentiated genes in T cell clusters in LPS+I3C versus LPS+Veh groups. (D) RORc and IL-22 mRNA expression in mononuclear cells (*n* = 3). (E) The Ingenuity Pathways Analysis tool predicted the top diseases and bio functions associated with the topmost dysregulated genes in T cell clusters when comparing LPS+I3C versus LPS+Veh. (F) IPA top networks linked to dysregulated genes in LPS+Veh versus LPS+I3C groups. (G) IPA pathway prediction graphical summary of the mRNAs involved with tissue morphology and cellular development as well as connective tissue development and function. The data are were depicted as the mean ± the standard error of the mean (SEM). Statistical significance was tested via a two-tailed, paired Student’s *t* test. In panel D: *, *P* = 0.0180; **, *P* = 0.0073.

To identify potential dysregulated genes in the T cell population when comparing the LPS+I3C versus LPS+Veh groups, scRNA-seq was performed on cells that were isolated from the lungs. The Loupe Browser was used for the downstream analysis of scRNAseq files to identify the fold change in dysregulated genes among various groups. Later, these data sets were fed into the Qiagen Ingenuity Pathways Analysis (IPA) application to identify the pathway predictions and molecular functions associated with the dysregulated genes in the lung cells of ARDS-induced mice with or without treatment with I3C (Fig. 6A). Among the top 50 genes dysregulated in single-cell T cell clusters of LPS-exposed mice versus LPS+I3C treated mice, IL-22 and RORc were among the top of this list (Fig. 6B and C). Notably, IL-22 expression was upregulated in LPS+I3C, compared to the LPS+Veh group. On the other hand, RORc expression was downregulated in the LPS+I3C group versus the LPS+Veh group (Fig. 6C). IL-22 and RORc expression were validated using qRT-PCR (Fig. 6D). Furthermore, the IPA detail-rich bioinformatic database linked the dysregulated cluster transcripts in the LPS+I3C versus LPS+Veh groups to the top diseases and bio functions involved in “molecular and cellular functions and physiological system development and function”. Interestingly, tissue morphology, cellular growth, and proliferation were the predicted canonical pathways that were identified to be affected following the I3C treatment of LPS-exposed animals (Fig. 6E). Notably, tissue morphology was also among the top networks predicted by IPA to be disturbed when comparing the LPS+I3C versus LPS+Veh groups (Fig. 6F). Lastly, to investigate the predicted relationship between the top dysregulated genes in the scRNAseq T cell clusters in the LPS+I3C versus LPS+Veh groups, network diagrams were generated using the IPA knowledge base network pathway tool. The IL-22 and RORc gene relationship findings were inconsistent with the state of the downstream regulation of each other, which may be due to RORc playing multiple roles in the development and inhibition of IL-22 producing cells (Th17 RORγt ^+^IL-17a^+^IL-22^+^ and Th22 IL-17 IL-22^+^) (Fig. 6G).

### miR-29b-2-5p targets genes associated with Th17/Th22 development.

**FIG 7:**
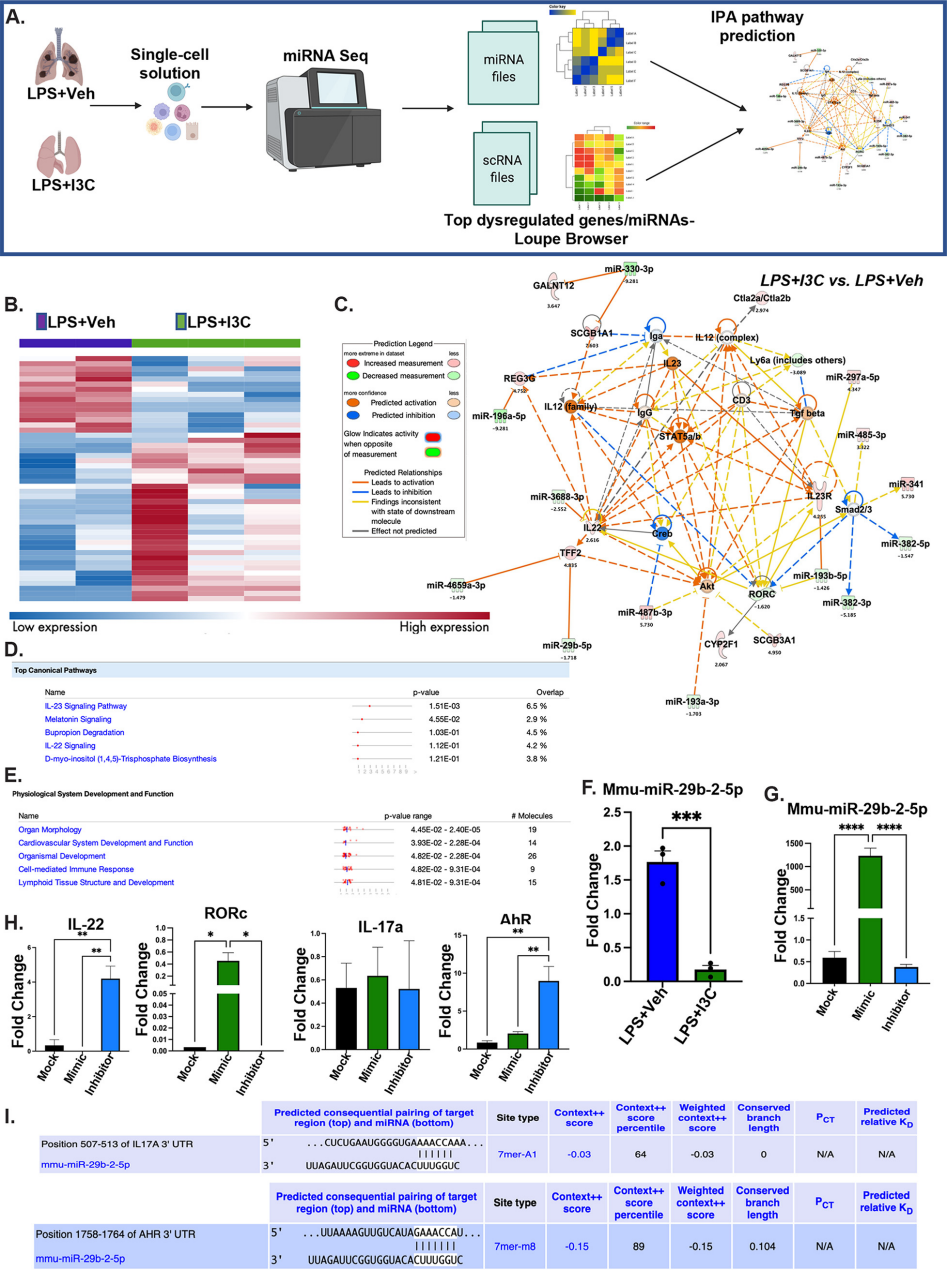
miR-29b-2-5p targets genes associated with IL-22 signaling. Mice were treated with LPS+Veh or LPS+I3C, as described in the legend of Fig. 1. The mice were euthanized at 48 h, and their lungs were processed for a miRNAseq analysis. (A) Experimental design schematic of the assays performed to identify the pathways associated with dysregulated genes in lung cells following LPS-exposure and I3C treatment using miRNAseq, scRNAseq, and IPA analyses. (B) Heat map of LPS+Veh versus LPS+I3C dysregulated miRNAs that were identified using miRNAseq technology. (C) Graphical summary of the IPA pathway prediction of miRNA dysregulation that was associated with the mRNAs that were predicted to be involved in IL-17a signaling in the airway, IL-22 signaling, and pulmonary healing signaling pathway functions when comparing the LPS+Veh versus LPS+I3C groups. (D and E) The top canonical pathways, top diseases, and biological functions associated with the highest dysregulated genes, as predicted by the Ingenuity Pathways Analysis tool, in T cell clusters when comparing the LPS+I3C versus LPS+Veh groups. (F) Expression of Mmu-miR-29b-2-5p in the treatment groups (*n* = 3). (G and H) A miRCURY LNA mimic and inhibitor assay was performed to validate the genes targeted by miR-29b-2-5p. The Lung MNCs of the C57BL6 mice were transfected with a mock, mimic, and inhibitor of miR-29b-2-5p. qRT-PCR was used to detect the levels of Mmu-miR-29b-2-5p and the targeted genes IL-22, IL-17a, RORC, and AhR (*n* = 4/technical replicate). (I) The miR-29b-2-5p specific gene targets that are associated with Th17/Th22 were identified using target scan software. The data are depicted as the mean ± the standard error of the mean (SEM). Statistical significance was tested via a two-tailed, one-way ANOVA or via a two-tailed, paired Student’s *t* test. In panel G: ****, *P* < 0.0001; ****, *P* < 0.0001. In panel H: **, *P* = 0.0025; *, *P* = 0.0016; *, *P* = 0.0291; *, *P* = 0.0282; **, *P* = 0.0017; **, *P* = 0.0068.

Lastly, we performed miRNA-seq using whole lungs to identify the dysregulated miRNAs that are associated with IL-22 and RORc expression in the LPS+I3C versus LPS+Veh groups. The miRNAseq data were concatenated with the scRNA-seq data and fed into IPA to predict the relationships between the top dysregulated genes and dysregulated miRNAs (Fig. 7A). A heat map was generated using the Qiagen RNA analysis portal, which showed an alteration in the miRNA profiles (≥1.5-fold increase or decrease in miRNA expression) in LPS+I3C versus LPS+Veh mice (Fig. 7B). Next, a network was generated using the IPA knowledge base network pathway tool using the miRNA and mRNA data sets of the LPS+I3C versus LPS+Veh groups to predict the relationships between dysregulated genes and miRNA. Notably, miR-3688-3p was the only miRNA that was - directly predicted to target IL-22, leading to its activation. miR-193b-5p was predicted to target RORc directly, but the findings were inconsistent with the state of the downstream regulation of the gene. miR-29b-2-5p and miR-4649a-3p were predicted to be closely but indirectly associated with IL-22, leading to its activation, whereas miR-341 and miR-382-5p were predicted to be closely but indirectly associated with RORc (Fig. 7C). Interestingly, the IPA knowledge base predicted “IL-22 signaling” as a top canonical pathway associated with the I3C-induced gene and miRNA dysregulation in LPS-exposed mice by applying a log [*P*-value] > 1.5 threshold. It was also noted that the top significantly dysregulated genes in the LPS+I3C group versus those of the LPS+Veh group were linked with several “physiological system development and function”, including “organ morphology” pathways (Fig. 7D and E). To validate the miRNAs associated with IL-22 and RORc, we performed qRT-PCR using MNCs. miR-29b-2-5p, a miRNA in the miR-29b-5p family, was the only dysregulated miRNA that was associated with IL-22 and RORc that we could validate, suggesting that miR-29b-2-5p may be specific to mononuclear cells, whereas the several other dysregulated miRNAs in relation to IL-22 and RORc may be restricted to cells other than lung mononuclear cells (Fig. 7F). Furthermore, a transfection assay using lung MNCs was performed to corroborate that the downregulation of miR-29b-2-5p increases the expression of IL-22 while downregulating the expression of RORc. Transfected cells were treated with a mock, mimic, or inhibitor of miR-29b-2-5p. qRT-PCR revealed that the miR-29b-2-5p specific mimic significantly increased the expression of miR-29b-2-5p and RORc but decreased the expression of IL-22 in transfected cells. In contrast, the miR-29b-2-5p specific inhibitor substantially increased the expression of IL-22 and AhR but decreased the expression of miR-29b-2-5p and RORc. The IL-17a expression was not altered in lung MNCs that were treated with the miR-29b-2-5p mimic or inhibitor, compared to cells treated with the mock (Fig. 7G and H). Interestingly, targetscan.org identified IL-17a and AhR as specific targets of miR-29b-2-5p (Fig. 7I). Furthermore, TFF2 gene expression was not changed in the MNCs and was not recognized as a specific target gene of miR-29b-2-5p when using the targetscan.org. IPA generated relationship connections between AhR, RORc, IL-17a, IL-22, and miR-29b-2-5p, which may explain why the RORc and IL-22 mRNA levels are altered following miR-29b-2-5p transfection assays when neither gene is a not direct target of the specific miRNA. miR-29b-2-5p altered the gene expression of AhR, which is the gene that codes for the AhR cytosolic transcription factor. The activation of the AhR transcription factor can upregulate IL-22 expression (31) and inhibit the expression of the Th17-related gene RORc (35). Taken together, these data suggest that miR-29b-2-5p may regulate the balance of Th17/Th22 subsets during acute lung injuries by altering AhR mRNA, which thereby alters the expression of IL-22 and RORc.

## DISCUSSION

ARDS is a life-threatening pulmonary disorder with a high mortality rate, and it currently has no approved FDA pharmacological treatment (36). The total annual health care cost for ARDS, due to hospitalization and intensive medical care, is more than $5 billion/year, and this cost is expected to increase due to the COVID-19 pandemic (37, 38). Sepsis, trauma, and certain viral and bacterial infections can cause ARDS, which induces the influx of proinflammatory immune cells in the alveolar space of the lungs (39). During ARDS, Th17 and Th17-related key transcription factors, namely, RORγt and the IL-17a cytokine, contribute to the disease severity (40). Studies have shown that increased levels of Th17 cells are found in the BALF of both mice and patients with lung diseases (41, 42). Furthermore, reports on IL-22, a cytokine produced by T cells, including Th17 cells, in ARDS have been contradictive, promoting the cytokine’s homeostasis and pathogenic roles (29, 43). IL-17a regulates the proinflammatory versus tissue-protective properties of IL-22, and in the absence of IL-17a, IL-22 is tissue-protective (29). IL-22 mediates tissue regeneration and epithelial repair following a lung injury (13). Interestingly, the AhR has been shown to play a role in the differentiation of Th17 (RORγt^+^ IL-17a^+^ IL-22^−^ and RORγt^+^ IL-17a^+^ IL-22^+^) cells and Th22 (IL-17a-IL-22^+^) cells (15, 31, 34, 35, 44–48). I3C, a natural aryl hydrocarbon receptor ligand, was reported to protect against inflammation by mediating the upregulation of IL-22 through the binding of the AhR on RORc-expressing immune cells (15). Furthermore, I3C induces the immunosuppressive behavior of immune cells and downregulates pathogenic Th17 in several inflammatory mouse models (49, 50).

Our study demonstrates that I3C alleviates LPS-induced ARDS severity by improving lung function and by maintaining lung structure integrity. I3C mediates this improvement through the regulations of the Th17/Th22 axis. In the LPS-induced ARDS model, the pathogenic Th17 cell population increased following ARDS induction, but the I3C treatment reduced the pathogenic Th17 cell population in LPS-exposed animals. A study by Li et al. (51) showed that suppressing the Th17 cell response protects mice from a lipopolysaccharide-induced lung injury. Furthermore, I3C increased the population of Th22 cells and IL-22 protein secretion in LPS+I3C versus LPS+Veh mice. Interestingly, during a lung infection, IL-22 plays an essential role in recovery. Treating LPS-induced ARDS mice with IL-22:FC recombinant protein decreased lung inflammation, dereased capillary protein leakage, prevented lung structural damage, and repaired ARDS-associated lung injuries (43). Interestingly, IL-22 is a cytokine secreted by Th17 cells, Th22 cells, γδ T cells, NKT cells, and innate lymphoid cells (ILCs) (52, 53). However, in our studies, the increase in the Th22 cell population and the reduction of pathogenic Th17 coincided with reduced lung tissue damage.

Furthermore, the I3C induction of Th22 cells was dependent on the RORγt^+^ cells that expressed AhR, inasmuch as the ablation of AhR on RORγt^+^ cells blocked an I3C-mediated increase in Th22 cells. Moreover, knocking out the AhR on RORγt^+^ cells prevented I3C tissue-protective properties. RORγt^+^ is encoded by the RORc gene, which is the master regulator of Th17 cells. Therefore, RORc inhibitors block Th17 differentiation and IL-17a production (54). As seen in our studies, RORγt^+^ is expressed by Th17 cells in both the RORγt^+^ IL-17a^+^ IL-22- and RORγt^+^ IL-17a^+^ IL-22^+^ phenotypes. During lung injury, the population of Th17 RORγt ^+^IL-17a^+^IL-22^+^ is increased. However, I3C decreases the expression of Th17 gene signatures (RORc and IL-17a) and increases Th22 gene signatures (AhR and IL-22) in polarized Th17 cells. Additionally, RORγt is also expressed on thymocytes, NK cells, γδ T cells, and ILCs (55–59). However, of these cells, ILCs are the only other population that co-express both AhR and RORγt^+^ and also secrete the IL-17a and IL-22 cytokines (15, 60, 61). Therefore, one limitation of this study is that the AhR deletion on RORγt^+^ cells was not isolated to only Th17 cells. However, we did not notice any changes in the population of ILCs expressing AhR and RORγt^+^ following ARDS induction and treatments. Furthermore, our studies also highlight the involvement of miRNAs that are associated with the shift in Th17 and Th22 cells following the treatment of mice with ARDS using I3C.

miRNA plays a critical role in regulating gene expression, especially regarding those that regulate the immune response (62, 63). Our lab has also previously shown that I3C attenuates staphylococcal enterotoxin B-mediated liver injury by downregulating miR-31 expression (64). I3C-mediated miRNA dysregulation was also shown in other inflammatory and cancer models (65, 66). In the current study, we performed scRNAseq and miRNAseq to look at the dysregulated mRNAs and miRNAs in whole lungs when comparing the LPS+Veh versus LPS+I3C groups of mice. We observed that treatment with I3C significantly altered the expression of several miRNAs that directly or indirectly targeted IL-22 and RORc, which is the gene for RORγt. Th17 differentiation and IL-17a production are dependent on RORc (54).

Using the concatenated miRNAseq and scRNAseq files, we used IPA pathway prediction to build a graphical summary of the dysregulated miRNAs that are associated with altered mRNA when comparing the LPS+Veh mice versus the LPS+I3C mice. miR-3688-3p was predicted to target IL-22 and thereby mediate its activation. On the other hand, miR-193b-5p was also predicted to target RORc. miR-29b-5p and miR-4649a-3p were predicted to be indirectly associated with IL-22, leading to its activation. miR-341 and miR-382-5p were predicted to be indirectly associated with RORc. Notably, after testing the several miRNAs targeting the RORc, AhR, and IL-22 genes, only miR-29b-2-5p, a miRNA in the miR-29b-5p family, was validated to target genes associated with Th17 and Th22 cells. Several miRNA members of the miR-29b-5p family are involved in regulating cellular proliferation, cellular differentiation, apoptosis, autoimmunity development, and fibrosis (67–70). Specifically, miR-29b-2-5p has been shown to regulate epithelial-mesenchymal transition progression in pulmonary fibrosis (71).

Isolated MNCs from the lungs of wild-type LPS+Veh and LPS+I3C mice showed that I3C downregulated miR-29b-2-5p. Transfection experiments using lung MNCs demonstrated that miR-29b-2-5p controls the Th17 and Th22 cell populations. A Mmu-miR-29b-2-5p mimic downregulated IL-22 and AhR gene expression, whereas a Mmu-miR-29b-2-5p power inhibitor had opposing effects. Conversely, a Mmu-miR-29b-2-5p mimic increased RORc expression, and a Mmu-miR-29b-2-5p power inhibitor had opposing effects on RORc expression. However, neither the Mmu-miR-29b-2-5p mimic nor the inhibitor altered IL-17a expression. However, using targetscan.org, we confirmed IL-17a and AhR to be specific target genes for miR-29b-2-5p. miR-29b-2-5p has binding affinity for both IL-17a and the AhR 3′UTR region, but it does not have binding affinity for IL-22 and RORc, suggesting that the alteration in their gene expression when treating ARDS with I3C may be a downstream effect of miR-29b-2-5p binding to IL-17a and/or AhR. For the differentiation of Th22 cells, AhR is an essential transcription factor, whereas RORc is a critical transcription factor for Th17 differentiation (54, 72). In a study of coronary heart disease, mimic transfection of miR-31 increased the levels of AhR and IL-22 and promoted Th22 cell differentiation (73). However, we did not see any changes in the expression of miR-31 across the treatment groups in our LPS-induced lung injury study.

Furthermore, the AhR-miR-29b-2-5p relationship had a context score of −0.15 and a context percentile score of 89%. On the other end, the IL-17a-miR-29b-2-5p relationship had a context score of −0.03 and a context percentile score of 64%. A more negative context score and a higher context percentile score indicated that a specific site or gene is more favorable than other sites of this miRNA with a higher context score and a lower context percentile score. This suggested that miR-29b-2-5p favors the AhR 3′UTR region over the IL-17a 3′UTR region, which may be an indicator of why we do not see a change in IL-17a expression in transfected MNCs when using a miR-29b-2-5p mimic or a miR-29b-2-5p power inhibitor.

In summary, the activation of AhR by I3C prevents excessive lung tissue damage by decreasing pathogenic Th17 cells and increasing Th22 cells. Upon looking at the mechanistic involvement of miRNAs in the I3C alleviation of ARDS, we observed that miR-29b-2-5p is involved in the Th17/Th22 axis. Data suggest that I3C downregulates miR-29b-2-5p in animals with ARDS, which in turn upregulates AhR expression, thereby leading to the downregulation of RORc expression and the upregulation of IL-22 in lung MNCs. IL-22 and AhR are gene signatures of Th22 cells, whereas RORc is a gene signature of Th17 cells. Our studies suggest that Th22 cells are associated with the alleviation of ARDS, whereas pathogenic Th17 cells are involved in the induction of the disease state. Thus, the activation of AhR by I3C leading to the attenuation of ARDS may be mediated by the involvement of miR-29b-2-5p and the Th17/Th22 axis.

### Limitations of the study.

Some innate lymphoid cells express AhR on RORγt+ cells that express IL-17A and IL-22. Thus, in our studies, AhR deletion on RORγt+ cells was not restricted to only Th17 cells. Furthermore, lung mononuclear cells were isolated from the lungs to perform a miRNA transfection experiment to compare the gene expression of IL-22 in immune cells. Under certain conditions, macrophages can express IL-22. Therefore, it is unclear whether the mmu-miR-29b-2-5p mimic and inhibitor regulated the IL-22 gene expression changes in macrophages or lymphocytes, although the transfection experiments showed the direct regulation of IL-22 by mmu-miR-29b-2-5p.

## MATERIALS AND METHODS

### Mice and ALI.

Female C57BL/6 and C3H HeJ mice, aged 8 to 10 weeks, were purchased from Jackson Laboratories (Bar Harbor, ME, USA). Female RORγtCreAhr^fl/fL^ on a C57BL/6 background were bred in-house. All mice were housed and maintained under specific pathogen-free conditions, under 12 h light/12 h dark cycles in temperature-controlled rooms, at the AAALAC-accredited animal facility at the University of South Carolina School of Medicine (Columbia, SC). Mice were given *ad libitum* access to water and a standard chow diet. To induce ARDS in mice, the animals were kept under light isoflurane anesthesia and oxygen for 5 min and then given 20 μL of LPS (2 mg/kg) or phosphate-buffered saline (PBS) intranasally. For the treatment groups, the mice were given 100 μL of I3C (60 mg/kg) or 100 μL of corn oil (vehicle) 3 h after the induction of disease. All treatments were suspended in corn oil and 10% DMSO. It should be noted that the doses of LPS and I3C were based on previous studies. Other studies have used LPS at doses of 1 to 5 mg/kg body weight to induce ARDS (74–76). In our standardizing experiments, we found that 2 mg/kg of LPS was a sufficient dose to induce significant ARDS. Also, previous findings from our laboratory showed that I3C was able to suppress colitis and delay hypersensitivity at doses of 40 to 50mg/kg body weight (15, 49). In the current study, we found that a single dose of 60 mg/kg was necessary and effective in alleviating ARDS in mice.

IL-22 neutralization was carried out as previously described by Busbee et al., (2020) (15). Briefly, C57BL/6 mice were injected intravenously with 50 mg of a rat IgG2a κ isotype control (eBR2a; 16-4321-85) or a functional grade anti-IL-22 monoclonal antibody (IL-22JOP; 16-7222-85; purchased from Thermo Fisher Scientific, Waltham, MA) 24 h prior to the administration of LPS (Escherichia coli, serotype 055:B5; Sigma-Aldrich, St. Louis, MO) and either a vehicle or an I3C treatment. The mice were given an additional 50 mg dose of the rat IgG2a κ isotype control or the functional grade anti-IL-22 monoclonal antibody immediately following treatment. After 48 h, all animals were euthanized.

### Histology.

Mouse lung histology for the detection of ARDS was carried out. Briefly, the mouse lungs were harvested and fixed in 4% paraformaldehyde for 24 h, and they were then placed in 70% ethanol for 24 h. They were eventually kept in 1% paraformaldehyde until they were embedded in paraffin. The embedded lungs were sectioned and processed for hematoxylin and eosin (H&E) staining. Later, the KEYENCE digital microscope VHX-7000 (Itasca, IL) was used to examine tissue injuries.

### Tissue processing.

At the experiment endpoint, the mice were euthanized, whole blood was collected from the portal veins of the mice, and serum was separated to perform enzyme-linked immunosorbent assays (ELISAs). In brief, to collect lung cells, euthanized mice were perfused with 10 mL of heparinized PBS. Lungs were excised, placed in 5 mL of flow cytometry staining buffer (FACS buffer-1× PBS, 2% fetal bovine serum [FBS], and 2 mM EDTA), homogenized into a single cell solution, and filtered with a 40 μM strainer. The cells were centrifuged at 1,300 rpm for 10 min at 4°C. The supernatant was discarded, and the cell pellet was resuspended in 1 mL of red blood cell (RBC) lysis buffer and placed on ice for 5 min. The single-cell solution was then neutralized with 5 mL of flow cytometry staining buffer (FACS) and centrifuged at 1,300 rpm for 5 min at 4°C. After discarding the supernatant, the cells were washed and centrifuged twice more. The cell pellet was resuspended in 5 mL FACS buffer and filtered. We used a Bio-Rad TC20 Automated Cell Counter (Hercules, CA) to count the cells in the single-cell suspension.

### ELISA.

Serum cytokines were detected using ELISA as described by Dopkins et al. 2021 (77). To detect cytokines in the serum, whole blood samples were centrifuged at 8,000 × *g* for 12 min, Afterwards, sera were collected and stored at −20°C. Lung MNCs were isolated from the lung single-cell solution that was previously described above, using the Sigma-Aldrich Histopaque-1077 (St. Louis, MO) density gradient centrifugation protocol to purify the MNCs as was previously described by Sultan et al. 2021 (78). The MNCs were plated at 1 × 10^6^ cells/well density in RPMI 1640 media supplemented with heat-inactivated 10% FBS, 10 mM l-glutamine, 10 mM HEPES, 50 μM β-mercaptoethanol, and 100 μg/mL penicillin/streptomycin for 24 h. ELISAs were performed to detect the IL-17a and IL-22 cytokine levels in the serum and in the culture supernatant.

### Polarization.

Th17 cell polarization was performed as described earlier by Becker et al. 2021 (79). To that end, spleens were processed into a single-cell suspension. CD4^+^ T cells were purified using an EasySep PE Positive Selection Kit (Stemcell Technologies, 18557; Cambridge, MA). The purity of the CD4^+^ T cells was validated using BD FACS Celesta. Samples with a >90% CD4^+^ T cell purity were considered to be a successful selection and were transferred into complete RPMI 1640 media (described above). The cells were plated at 1 × 10^6^ cells/mL density in 12-well plates that were coated with CD3ε, clone 145-2C11 (3 μg/mL), and treated with anti-mouse CD28, clone 37.51 (3 μg/mL). After 48 h, to polarize toward Th17, the cells were incubated with recombinant IL-1β (10 ng/mL), IL-6 (30 ng/mL), IL-23 (20 ng/mL), and FICZ (400 nM) for 3 days. Flow cytometry and ELISAs were performed to validate the Th17 polarization.

### Locked nucleic acid transfection.

Lung MNCs were used to perform miRNA transfection. In brief, as described previously (78), lung MNCs were isolated using the Sigma-Aldrich Histopaque-1077 (St. Louis, MO) density gradient centrifugation protocol. The cells were plated in a 24-well plate at 250,000 cells/well density in RPMI 1640 media that was supplemented with heat-inactivated 10% FBS, 10 mM l-glutamine, 10 mM HEPES, 50 μM β-mercaptoethanol, and 100 μg/mL penicillin/streptomycin. As previously described [36], the cells were transfected with either 2.5 pmol mock control, 2.5 pmol synthetic mimic mmu-miR-29b-2-5p (CUGGUUUCACAUGGUGGCUUAGAUU), or 25 pmol mmu-miR-29b-2-5p (TAAGCCACCATGTGAAACCA) power inhibitor purchased from Qiagen (Valencia, CA), using the Qiagen HiPerfect transfection reagent. qRT-PCR was used to validate the transfection efficiency. The validation of the expression levels of miR-29b-2-5p, IL-17a, IL-22, RORc, and AhR in the transfected cells was performed using qRT-PCR.

### Flow cytometry.

A flow cytometry analysis was performed on the lung cells. To analyze the Th17/Th22 subsets, 1 × 10^6^ cells/mL were incubated with TruStain FcX anti-mouse CD16/32 (San Diego, CA) for 10 min at room temperature, and the cells were subsequently incubated with the fluorescently labeled monoclonal antibodies (MAbs) anti-CD45 (APC/Cy7), anti-CD3 (BV7856, PE, or FITC), anti-CD4 (APC/Cy7, PE, or BV786), anti-LIN (AF700), and NKP46 (BV510), purchased from Biolegend, for 30 min on ice. BD Cytofix/Cytoperm (BD Biosciences, Franklin Lakes, NJ) was used to fix and permeabilize the cells for intracellular staining using anti-IL-22 (AF647), anti-IL-17a (BV510 or FITC), and RORγt (PE, BV650, or APC). To determine the cell percentages of the Th17/Th22 cells, BD FACS Celesta and FlowJo v10 software were used. The percentages of Th17/Th22 were multiplied by the total number of cells isolated from the lungs to determine the absolute cell counts of the populations.

### Quantitative-real time PCR (qRT-PCR).

cDNA was synthesized from whole lung RNA and from the RNA of transfected MNCs using a miScript II RT Kit from Qiagen. SsoAdvanced SYBR Green Supermix from Bio-Rad (Hercules, CA), along with sample cDNA and the mouse primers IL-17a (forward primer: 5′-CAGACTACCTCAACCGTTCCAC-3′ and reverse primer: 5′-TCCAGCTTTCCCTCCGCATTGA-3′), IL-22 (forward primer: 5′-GCTTGAGGTGTCCAACTTCCAG-3′ and reverse primer: 5′-ACTCCTCGGAACAGTTTCTCCC-3′), RORc (forward primer: 5′-GTGGAGTTTGCCAAGCGGCTTT-3′ and reverse primer: 5′-CCTGCACATTCTGACTAGGACG-3′), and AhR (forward primer: 5′-GCGGCCGCAGGAAGTGAGG-3′ and reverse primer: 5′GTGCCGTTGATTTGCGTGTGCT-3′), was used to conduct qRT-PCR to validate the mRNA expression.

A Qiagen miRCURY LNA RT Kit was used to synthesize cDNA from miRNA that was isolated from the whole lung and transfected MNCs. For the miRNA validation, qRT-PCR was performed using a miScript SYBR Green PCR Kit and miR-29b-2-5p mouse primer purchased from Qiagen (Valencia, CA). GAPDH and Snord96A were used as endogenous controls for the cDNA that was generated from the RNA and miRNA.

### Single-cell RNA sequencing (scRNAseq).

The lungs of the mice that were euthanized 48 h after treatment with LPS+Veh or LPS+I3C were processed for an scRNAseq analysis. The lungs were excised and pooled in 5 mL of FACS buffer-1× PBS, 1% fetal bovine serum (FBS), and 2 mM EDTA. Then, they were homogenized into a single cell suspension and filtered with a 40 μM strainer. Next, the single-cell suspension was centrifuged at 1,300 rpm for 10 min at 4°C, and the supernatant was discarded. The cells were resuspended in 1 mL of RBC lysis buffer and placed on ice for 5 min, and the single-cell suspension was then treated with 5 mL of FACS buffer and centrifuged at 1,300 rpm for 5 min at 4°C. After discarding the supernatant, the cells were washed and centrifuged twice more. The cell pellet was resuspended in 5 mL FACS buffer and filtered. Following the cell resuspension, we used a TC20 Automated Cell Counter [Bio-Rad] to measure the cell number and viability. The samples were processed with a Stem Cell Technology Dead Cell Removal Kit (Cambridge, MA) to ensure that all samples had >70% viability. Following the 10× genomics protocol, 3,000 target cells per sample (Vehicle, LPS+Veh, LPS+I3C) were loaded into separate lanes on a 10× Genomics Chromium Controller. Using the manufacturer’s protocol exclusively for gene expression, single-cell RNA-seq libraries were generated from the samples using a Chromium v2 Single-cell 5′ VDJ RNA-seq Reagent Kit (10× Genomics). Then, sequencing followed, using an Illumina NextSeq 550, targeting a read depth of at least 20,000 to 30,000 reads per cell. The raw base call files that were generated from the sequencer were then analyzed using the 10× Genomics Cell Ranger software package [version 2.0] to generate FASTQ, Count, and cloupe files for further processing. A downstream analysis was performed using the 10× Genomics Loupe Browser version 5.1 software package to visualize and compare the single-cell gene expression across generated sample clusters, and this was illustrated via *t*-distributed stochastic neighborhood embedding (tSNE) plots.

### microRNA sequencing.

Following the manufacturer’s instructions, a MiRNeasy Minikit (Qiagen, Hilden, Germany) was used for RNA extraction, and the quantification of total RNA was extracted from samples that contained at least 1 × 10^6^ cells/mL. Afterward, a Qubit High Sensitivity RNA Assay Kit (Thermo Fisher, Waltham, MA) was used to confirm the RNA concentrations of the samples. Next, using 100 ng of miRNeasy RNA, miRNA libraries were prepped using a QIAseq miRNA Library Kit (Qiagen), following the manufacture’s protocols. A 1.5% agarose gel was used to quantify the size for each library, and this was followed by the sequencing of the libraries on a NextSeq 550 Sequencer (Illumina), using version 2 chemistry. Afterward, FASTQ files were uploaded to the GeneGlobe tool (Qiagen), the data were aligned, and hit counts were created using the RNA-seq Analysis Portal. For the secondary analysis, samples were queued, in which the miRNAs from all samples were normalized and the fold change was resolved. MiRNAs with false discovery rate (FDR) *P*-values of <0.05 and fold changes of <−1.5 or >1.5 were selected for further investigation.

### Evaluation of lung function.

Whole-body plethysmography (Buxco, Troy, NY) was used to measure the lung functioning of the experimental mice. The plethysmography calculated the clinical parameters, including the rejection index (RINX), tidal volume (TV), ratio of peak expiratory flow (Rpef), frequency (f), specific airway resistance (sRAW), specific airway conductance (sGAW), and minute per volume (MV).

### Statistical analysis.

The number of mice used is depicted in each figure. GraphPad Prism Version 9.000 for Mac (GraphPad Software) was used for the statistical analyses. To compare data between two groups, a Student’s *t* test was used. A one-way analysis of variance (ANOVA) with a Tukey’s *post hoc* test was performed when comparing three or more groups. The Shapiro-Wilk test was used before the Student’s *t* test and the one-way ANOVA to check for normality before the comparison was conducted. All of the values are presented as a mean ± the standard error of the mean (SEM), and a *P* value of <0.05 was considered to be indicative of a statistically significant result.

### Data availability.

The data presented in this publication have been deposited in NCBI's Gene Expression Omnibus and are accessible through the GEO Series accession number, GSE224938.
